# Sulforaphane Inhibits IL-1β-Induced IL-6 by Suppressing ROS Production, AP-1, and STAT3 in Colorectal Cancer HT-29 Cells

**DOI:** 10.3390/antiox13040406

**Published:** 2024-03-28

**Authors:** Dhiraj Kumar Sah, Archana Arjunan, Seon Young Park, Bora Lee, Young Do Jung

**Affiliations:** 1 Department of Biochemistry, Chonnam National University Medical School, Hwasun 58128, Republic of Korea; 197784@chonnam.edu (D.K.S.); archanaibms@gmail.com (A.A.); 2 Department of Internal Medicine, Chonnam National University Medical School, Gwangju 501190, Republic of Korea; drpsy@jnu.ac.kr

**Keywords:** colorectal cancer, sulforaphane, MAPK, ROS, IL-1β, IL-6, AP-1

## Abstract

Colorectal cancer (CRC) stands as a major cause of cancer-related mortality globally, accounting for approximately 881,000 deaths each year. Traditional approaches such as chemotherapy and surgery have been the primary treatment modalities, yet the outcomes for patients with metastatic CRC are often unsatisfactory. Recent research has focused on targeting the pathways involved in oxidative stress, inflammation, and metastasis to enhance the survival of CRC patients. Within this context, sulforaphane (SFN), a notable phytochemical found predominantly in cruciferous vegetables, has been recognized as a potential anticancer agent. However, the specific mechanisms through which SFN may exert its chemopreventive effects in CRC remain unclear. This study explores the impact of SFN on IL-1β-induced IL-6 activation and MAPK and AP-1 signaling in HT-29 cells. Our findings reveal that SFN treatment not only diminishes IL-1β-stimulated IL-6 expression but also reduces oxidative stress by curtailing reactive oxygen species (ROS) production. Furthermore, it hinders the proliferation and invasiveness of HT-29 cells through the modulation of MAPK/AP-1 and STAT3 signaling pathways. These results indicate that SFN mitigates IL-1β-induced IL-6 expression in CRC cells by attenuating ROS production and disrupting MAPK/AP-1 signaling. This suggests that SFN holds significant potential as a chemotherapeutic agent for both treating and preventing CRC.

## 1. Introduction

Cancer is characterized by a multi-stage aberrant signaling pathway resulting from the uncontrolled proliferation of transformed cells. Colorectal cancer (CRC; colon and/or rectal) ranks as the third most commonly diagnosed and the second most deadly cancer globally [1]. By 2035, the mortality rates due to colon and rectal cancer are projected to increase by 71.5% and 60%, respectively [2]. CRC arises from the aberrant proliferation of glandular epithelial cells in the colon and can be hereditary, sporadic, or linked to colitis [3]. The development of CRC involves both genetic and environmental factors [4]. Furthermore, individuals with ulcerative colitis and Crohn’s disease face a heightened risk of CRC with advancing age [5]. Several studies have demonstrated that diet [6,7], lifestyle [8,9], family history [10,11], and chronic inflammation [12,13] are risk factors for developing CRC.

Chronic inflammation plays a role in the development of various cancers, including CRC [14]. Notably, inflammatory mediators are present in all tumors, even those not arising from chronic inflammation [15]. The tumor’s inflamed microenvironment, often considered the seventh hallmark of cancer, promotes tumor progression [16]. Interleukin (IL)-1β, secreted by stromal, immune, and tumor cells, is a critical mediator of cancer-related inflammation [17]. Elevated levels of IL-1β have been observed in various cancers, including colon cancer, one of the most fatal [18,19,20]. Recent studies indicate that the interaction between immune cells and colon cancer cells leads to the increased secretion of IL-1β by immune cells, correlating with enhanced CRC invasion and growth [21,22,23]. However, the precise role of IL-1β in cancer initiation remains insufficiently explored.

The induction of IL-1β can lead to the release of several proinflammatory cytokines, which significantly influence tumor development. Among these, IL-6 plays a central role in human cancer progression [24]. Notably, IL-6 expression is associated with a poor prognosis in various types of cancer, including sporadic and colitis-associated cancers [25]. IL-6 activates multiple signaling pathways, such as the IL-6/STAT3 pathway [26] and mediates the generation of reactive oxygen species (ROS). These ROS activate IL-6/STAT3 signaling [27]. Additionally, IL-6 can activate the mitogen-activated protein kinase (MAPK), phosphoinositide 3-kinase (PI3K), and activator protein-1 (AP-1) pathways [28,29], which contribute to the proliferation and survival of cancer cells. Recent years have seen the development of several therapeutics targeting the IL-6/STAT3 pathway, offering a promising strategy for treating CRC. In this study, we elucidate the molecular mechanisms of IL-6 signaling in CRC, aiming to identify novel therapeutic approaches.

Therapeutic interventions for advanced or metastatic CRC have seen significant advancements recently. This has led to the emergence of novel drugs that primarily target oxidative stress-induced inflammatory responses. These drugs aim to counteract aberrant cancer signaling, growth, and proliferation, providing oncologists with advanced options for combating late-stage CRC. Numerous studies have explored the antitumor properties of natural product extracts in various cancers, focusing on mechanisms like proliferation, immune modulation, autophagy, and apoptosis. Sulforaphane (SFN), belonging to the isothiocyanate (ITC) group and an organosulfur compound is predominantly found in cruciferous vegetables. Research has shown that SFN possesses a wide range of activities, exhibiting potential as an antioxidant [30], antitumor [31], antiangiogenic [32], and anti-inflammatory [33] compound. SFN has been shown to inhibit the proliferation and promote apoptosis in CRC cells through various mechanisms. However, the effects of SFN on ROS-mediated MAPK/AP-1 expression in CRC are not yet fully understood.

## 2. Materials and Methods

### 2.1. Bioinformatics Analysis

We employed the Gene Expression Profiling Interactive Analysis (GEPIA) database to assess IL-1β expression in CRC samples using the GEPIA2 public database (http://gepia2.cancer-pku.cn/ (accessed on 2 February 2024)), which includes data from 275 tumors and 349 normal samples. GEPIA, an online tool providing data concerning gene expression, tumor stage/grade, and survival is widely adopted to compare the gene expression between tumor and normal tissues, based on the Cancer Genome Atlas (TCGA) and the Genotype-Tissue Expression (GTEx). To investigate the correlation and network interactions between IL-1β and the genes IL-6, MAPK1, STAT3, and AP-1, we utilized TIMER2.0 (http://timer.cistrome.org/ (accessed on 2 February 2024)).

### 2.2. Cell Culture

We acquired sulforaphane (≥95% HPLC), N-acetylcysteine (NAC), dimethyl sulfoxide (DMSO), and 3-[4,5-dimethylthiazol-2-yl]-2,5-diphenyltetrazolium bromide from Sigma-Aldrich Co. (St. Louis, MO, USA). IL-1β, sourced from R&D Systems (Minneapolis, MN, USA) was collected at various intervals during the experiment. Inhibitors PD, SP, and SB203580 were obtained from Calbiochem (San Diego, CA, USA), while Stattic (a Stat3 inhibitor) came from Sigma-Aldrich (USA). HT-29 human colon cancer cells, provided by the American Type Culture Collection (Rockville, MD, USA) were cultured in Dulbecco’s Modified Eagle Medium (DMEM) with 10% fetal bovine serum (FBS) and 1% penicillin-streptomycin at 37 °C in a CO_2_ atmosphere. The chemicals were dissolved in DMSO and added to the culture media as stock solutions. We established a control group treated only with DMSO, ensuring the final DMSO concentration was below 0.1%.

### 2.3. Western Blot Analysis

For protein extraction from HT-29 cells (5 × 10^5^/mL) cultured in a 60 × 15 mm cell culture dish (SPL Life Sciences, Gyeonggi-do, Republic of Korea), we used the Pro-PREPTM protein extraction solution (150 μL/plate) (iNtRON Biotechnology, Gyeonggi-do, Republic of Korea). Materials like the polyvinylidene fluoride membrane and Western chemiluminescent HRP substrate were procured from Millipore Corporation (Billerica, MA, USA). We separated 30 μg of total protein using 10% sodium dodecyl sulfate–polyacrylamide gel electrophoresis and transferred it to polyvinylidene fluoride membranes. The membranes were blocked for 1 h with 0.1% Tween-20 in TBST containing 5% skim milk, followed by overnight incubation with primary antibodies (1:1000) in TBST at 4 °C. After four TBST washes of 10 min each, we employed horseradish peroxidase-conjugated secondary antibody (1:5000) for the detection of immunoreactive proteins via chemiluminescence. We utilized various antibodies from Cell Signaling Technology (Danvers, MA, USA) and Santa Cruz Biotechnology (Santa Cruz, TX, USA) for the Western blot experiments, including anti-phospho-Erk1/2 (Cell Signaling, #92552), anti-phospho-c-Fos (Santa Cruz, sc-81485), anti-c-Fos (Santa Cruz, sc-7202), anti-phospho-c-Jun (Cell Signaling, #3270S), anti-c-Jun (Santa Cruz, sc-44), anti-p44/42 MAPK (ERK1/2) (Cell Signaling, #9102S), anti-phospho-p38 (Cell Signaling, #9211L), anti-p38 (Cell Signaling, 9212S), anti-phospho-JNK (Cell Signaling, #4668), anti-JNK (Cell Signaling, #9255L), anti-phospho-STAT-3 (Cell Signaling, #9145S), anti-STAT-3 (Cell Signaling, #4904S), and anti-β-actin (Cell Signaling, 5125S) monoclonal antibodies. To evaluate total protein levels, the blotted membranes were washed for 30 min at 56 °C in RestoreTM Western Blot Stripping buffer (Thermo Fisher Scientific, Meridian Rd., Rockford, IL, USA).

### 2.4. Reverse Transcription Polymerase Chain Reaction (RT-PCR)

Total RNA was extracted from HT-29 cells (5 × 10^5^/mL) cultured in 6 well plates (SPL Life Sciences) using TRIzol reagent 400 µL per well (Invitrogen, Waltham, MA, USA) according to the manufacturer’s instructions. We then used 1 µg of this total RNA for the synthesis of first-strand complementary DNA, utilizing random primers (Promega, Madison, WI, USA) and M-MLV transcriptase (Promega). For amplifying the complementary DNA, we employed a PCR master mix solution (iNtRON, Republic of Korea) with primer sets specific for β-actin and IL-6. The primers used were: β-actin forward (accession number: NM_001101.5, NCBI Reference Sequence), 5′-AAG CAG GAG TAT GAC GAG TC-3′ and β-actin reverse, 5′-GCC TTC ATA CAT CTC AAG TT-3′ (561 bp); IL-6 forward (accession number: NM_001371096.1, NCBI Reference Sequence), 5′-ACA CAG ACA GCC ACT CAC C-3′ and IL-6 reverse, 5′-TACATTTGCCGAAGAGCC-3′ (513 bp) [34]. The primer sequences are listed in Supplementary Table S1.

### 2.5. Real-Time Quantitative Polymerase Chain Reaction (RT-qPCR)

We utilized the same random primer as in RT-PCR for synthesizing the first strand of complementary DNA. The RT-qPCR was conducted using the FastStartTM SYBR Green Master Kit (Applied BiosystemsTM, Thermo Fisher, Foster City, CA, USA). The primers for RT-qPCR included: GAPDH forward (accession number: NM_001289745.3, NCBI Reference Sequence), 5′-TGG TAT CGT GGA AGG ACT CA-3′; GAPDH reverse, 5′-GGA TGA TGT TCT GGA GAG CC-3′ [35]; IL-6 forward (accession number: NM_001371096.1, NCBI Reference Sequence), 5′-ACA CAG ACA GCC ACT CAC C-3′ and IL-6 reverse, 5′-TACATTTGCCGAAGAGCC-3′. The primer sequences are listed in Supplementary Table S1.

### 2.6. Analysis of Matrigel Invasion

For the cell invasion assay, we employed a Corning Matrigel Invasion Chamber 24 well plate 8.0 Micron (Corning Inc., Steuben, NY, USA). The chemoattractant, DMEM containing 10% FBS was placed in the lower chamber. In the upper chamber, we introduced a layer of HT-29 cells (10^5^ cells in 300 µL) and allowed them to invade the Matrigel for 24 h, either with or without prior treatment with SFN and IL-1β. To determine the effects of signaling inhibitors on IL-1β cell invasion, HT-29 cells were preincubated with various signaling inhibitors for one hour and incubated with IL-1β for 24 h. We conducted two processes on the upper surface of the membrane: removal of non-invading cells and staining of the invading cells on the lower surface with a Quick-Diff Stain Kit (Sysmex Corporation, Kobe, Japan), following the manufacturer’s protocol. After washing the chambers twice with phosphate-buffered saline (PBS), the invading cells were counted using a phase-contrast microscope.

### 2.7. Analysis of Promoter Activity

Next, we performed transient transfection using a promoter-luciferase reporter construct (pGL3-IL-6) to investigate the transcriptional regulation of IL-6. The plasmid (pGL3-IL-6, pGL3-AP-1) was kindly provided by Dr Yoon (Konkuk University, Seoul, Republic of Korea). HT-29 cells were seeded and grown until reaching 70% confluency and were transfected with the pGL3-IL-6 promoter plasmid using FuGENE (Promega, USA) according to the manufacturer’s protocol and pRL-TK which served as the internal control. The cells underwent pre-treatment with various inhibitors for one hour prior to IL-1β addition for 12 h to evaluate the impact on IL-6-promoter activity. Additionally, co-transfection experiments were conducted both with and without SFN, signaling inhibitors, and a dominant-negative mutant of the p38 gene (p38-DN), Erk (K97), and JNK (TAM), to investigate the influence of these factors on IL-1β-induced IL-6 expression These expression vectors were gifted by Dr N.G. Ahn (University of Colorado Boulder), Dr M.J. Birrer (University of Helsinki), and Dr Jiahuai Han (Scripps Research Institute), respectively. The cells were harvested with a passive lysis buffer, and luciferase activity was determined using the Dual-LuciferaseTM Reporter Assay System (Promega) with a Centro LB 960 Microplate Luminometer (Berthold Technology, Bad Wildbad, Germany) according to the manufacturer’s protocol.

### 2.8. Measurement of Intracellular Hydrogen Peroxide (H_2_O_2_)

The concentration of intracellular H_2_O_2_ was assessed using 5- and 6-amino-5- and 6-amino-6-amino-2′,7′-dichlorodichlorofuoresceindiacetate (DCFDA; Molecular Probes, Eugene, OR, USA). Briefly, HT-29 cells (5 × 10^5^/mL) cultured in 6 well plates (SPL Life Sciences) were grown in DMEM supplemented with 10% FBS to 80% confluence, then washed with PBS, and transferred to serum-free DMEM for 12 h. To evaluate the effects of IL-1β on ROS production, the cells were treated with 10 μM SFN or 1 mM NAC one hour before the IL-1β administration. Following this, the cells were incubated with 10 μM DCFDA for 15 min and then examined using a laser scanning confocal microscope (Carl Zeiss, Jena, Germany). DCFDA fluorescence was excited at 488 nm with an argon laser and emission at 515 nm was captured using a longpass filter. After incubation with DCFDA, the cells were examined in BD FACS Calibur (BD Biosciences, Franklin Lakes, NJ, USA) for the flow cytometry analysis for ROS. The data were plotted and subjected to analysis using FlowJo™ Software version 10.10, (BD Biosciences, San Jose, CA, USA).

### 2.9. Measuring IL-6 Secretion

HT 29 cells (2 × 10^5^ cells/well) were cultured in DMEM medium with 10% FBS at 37 °C in a 12-well plate (SPL Life Sciences). After a 24-h incubation period, the cells underwent a medium change to a fresh one with 1% FBS and were left to incubate overnight. The cells were treated by IL-1β for 24 h and Sulforaphane (1–10 µM) 1 h prior to IL-1β treatment. Cell culture supernatants were collected, and the IL-6 cytokine secreted by the cells was measured using an ELISA kit specifically designed for IL-6 (BD Bioscience). The IL-6 cytokine in the culture supernatants was assessed with a microplate spectrophotometer (Bio Tek Instruments, Winooski, VT, USA) and levels were calculated by matching their optical densities with values on the standard curve, as per the manufacturer’s instructions; the results are presented in pg/mL.

### 2.10. Statistical Analysis

Each value in this study is derived from three independent experiments and is expressed as mean ± standard deviation (SD). Data visualization was conducted using GraphPad Prism software (Version 8.0). For multivariate analysis, ANOVA with Tukey’s multiple comparison test was employed; a *p*-value of less than 0.05 (#, *) was considered statistically significant.

## 3. Results

### 3.1. IL-1β Expression and Correlation in CRC

**Figure 1:**
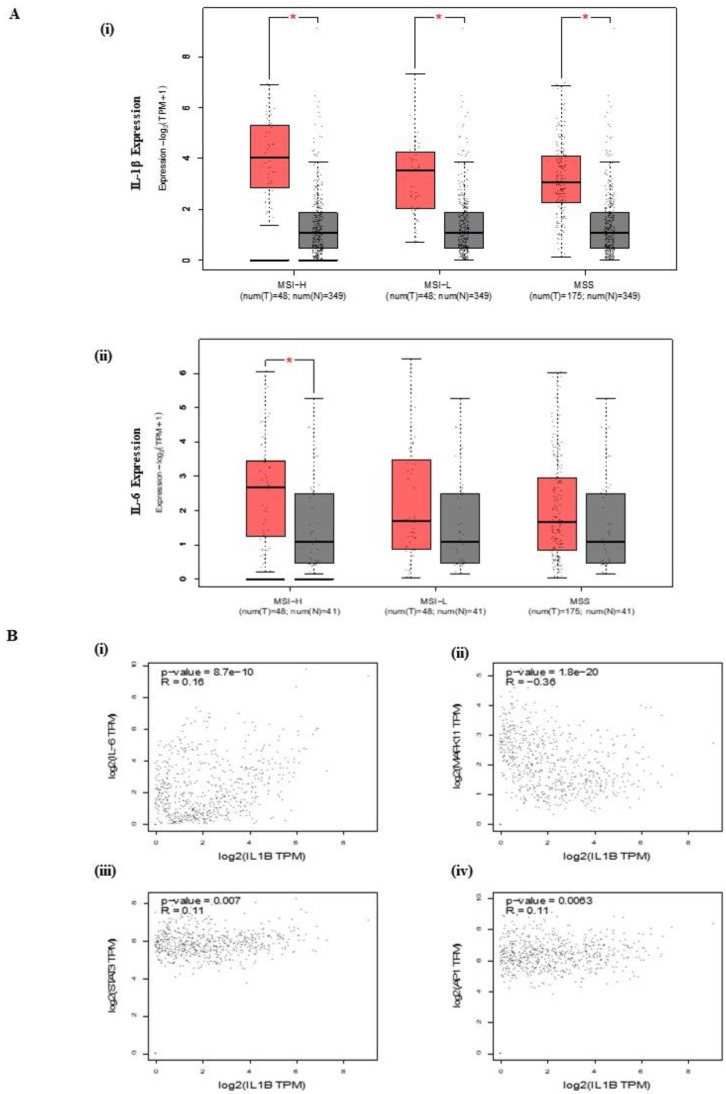
Interaction and expression of inflammatory markers in COAD. (**A**) Expression levels of (i) IL1B (Microsatellite instability-High (MSI-H) (num(T) = 48; num(N) = 349), Microsatellite instability-Low (MSI-L) (num(T) = 48; num(N) = 349), Microsatellite stable (MSS) (num(T) = 175; num(N) = 349)) and (ii) IL-6 in COAD, showing statistical significance (* *p* < 0.05). (**B**) Analysis of expression correlation and protein interactions between IL-6 (i), MAPK1 (ii), STAT3 (iii), and JUN (iv), with IL1B in COAD, utilizing data from TIMER2.0 (*p* < 0.05). (T—Tumor; N—Normal; num—Number).

To evaluate the accuracy of IL-1β and IL-6 expression, biopsies of COAD cancer type vs. the matched healthy tissue were used, with a statistical significance set at *p* < 0.05, according to the tool: Expression analysis/Expression DIY/Box plot in the GEPIA2 database. Figure 1A illustrates the log-transformed expression levels of IL1B and IL-6 in Colon adenocarcinoma (COAD), revealing a notable increase in these expressions. The IL1B expression in a normal colon is 1.13 Transcripts Per Millions (TPM), and COAD is 8.28 TPM. The IL1B expression (log2 (TPM + 1)) in COAD is significantly increased (*p* = 0.01). The expression DIY analysis of IL1B expression in all three subtypes (MSH-H, MSH-L, and MSS) of COAD significantly increased when compared to normal samples. The pair-wise gene expression correlation analysis for given sets of TCGA and/or GTEx expression data was performed using the Kendall method (Figure 1B). Additionally, Figure 1B presents a significant correlation between the expressions of IL-6, STAT3, and MAPK1 with IL-1β, as shown by TIMER2.0 analysis.

### 3.2. SFN Inhibits IL-1β-Induced IL-6 mRNA Expression in HT-29 Cells

**Figure 2:**
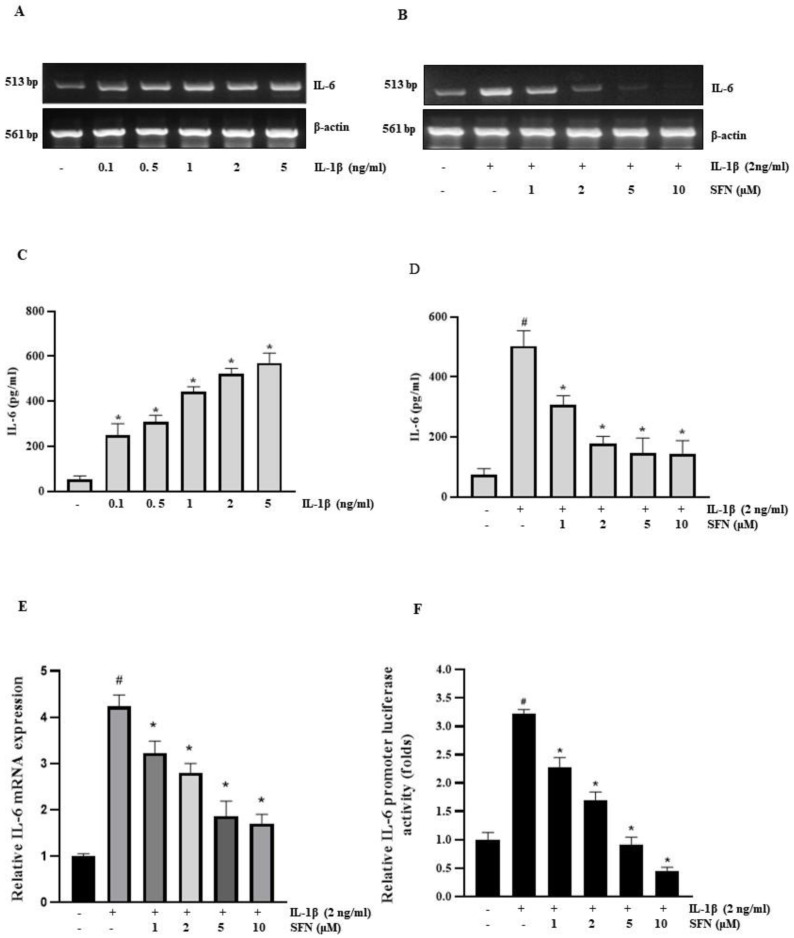
Sulforaphane inhibits IL-1β-induced IL-6 in HT-29 cells. (**A**) CRC cell line, HT-29 was treated with 0–5 ng/mL IL-1β for 4 h, followed by mRNA extraction and IL-6 expression analysis via RT-PCR. (**B**) Cells were pre-treated with 1–10 µM sulforaphane for 1 h, then exposed to 2 ng/mL IL-1β for 4 h. IL-6 mRNA levels were assessed by RT-PCR. (**C**) HT-29 cells were treated with IL-1β (0.1–5 ng/mL) for 24 h after that ELISA was performed to check the secreted IL-6, (**D**) Cells were pretreated with Sulforaphane (1, 5 and 10 µM) and incubated with 2 ng/mL IL-1β for 24 h, followed by ELISA assay to determined the secreted IL-6 level. (**E**) RT-qPCR analysis to determine the IL-6 expression level in HT-29 cells pretreated with (1–10 μΜ) Sulforaphane and incubated with 2 ng/mL IL-1β for 4 h. (**F**) Cells transiently transfected with 1 µg of pGL3-IL-6-promoter reporter construct were pre-treated with sulforaphane for 1 h. After 12-h incubation with 2 ng/mL IL-1β, luciferase activity was measured using a luminometer. Significance markers: # *p* < 0.05 versus control; * *p* < 0.05 versus IL-1Β. Data represent mean ± SD from triplicate experiments.

We assessed the inhibitory effect of SFN on IL-1β-induced IL-6 expression in HT-29 human colorectal cell lines using RT-PCR and ELISA. After pre-treating cells with SFN for 1 h, we administered IL-1β for 4 h. Following this, we measured IL-6 expression via RT-PCR after extracting total mRNA. The cells were pre-treated with 0–10 µM Sulforaphane, then exposed to 2 ng/mL IL-1β for 4 h. Both RT-PCR and Q-PCR were employed to assess IL-6 transcription levels. Figure 2B,E show that SFN pre-treatment significantly inhibited the upregulation of IL-6 induced by IL-1β in a dose-dependent manner. Moreover, as can be seen in Figure 2C,D, using the human-specific IL-6 ELISA assay IL-1β elevated the secreted IL-6 level in HT 29 cells, whereas Sulforaphane inhibited IL-1β-induced secreted IL-6 level in dose-dependent manner. The IL-6 promoter luciferase assay further confirmed the inhibitory effect of SFN on IL-1β-stimulated promoter activity using a luminometer, demonstrating a dose-dependent decrease in activity (Figure 2F).

### 3.3. SFN Suppresses IL-1β-Induced IL-6 by Inhibiting the p38 MAPK Pathway

**Figure 3:**
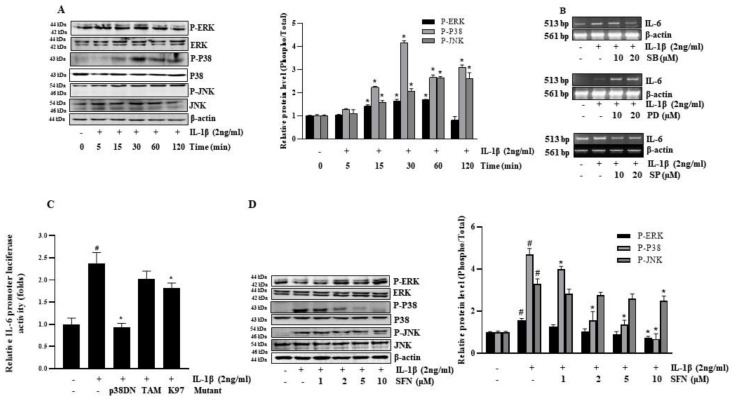
Sulforaphane inhibits IL-1β-induced IL-6 via suppression of p38 MAPK in HT-29 cells. (**A**) Cells were treated with IL-1β for 0–120 min, and phosphorylation of ERK1/2, JNK1/2, and p38 MAPK was analyzed by Western blotting. (**B**) RT-PCR assessed IL-6 mRNA levels in cell lysates following 1-h pre-treatment with varying concentrations of PD98059 (PD), SP600125 (SP), and SB203580 (SB) and 4-h incubation with IL-1β. (**C**) Cells cotransfected with dominant-negative mutant plasmids of JNK (TAM), ERK1/2 (K97M), and p38 MAPK (p38-DN) (1 µg each) and 1 µg of pGL3-IL-6 promoter plasmid were subjected to luciferase assays after 12 h of 2 ng/mL IL-1β treatment. (**D**) HT-29 cells were treated with 1–10 µM sulforaphane for 1 h before a 30-min exposure to 2 ng/mL IL-1β. Levels of phosphorylated ERK1/2, JNK, and P-38 in the cells were then analyzed. Significance markers: # *p* < 0.05 versus control; * *p* < 0.05 versus IL-1β. Data represent mean ± SD from triplicate experiments.

This study utilized the HT-29 colon cancer cell line to explore MAPK signaling’s role in IL-1β-induced IL-6 expression. Figure 3A illustrates that IL-1β triggers the activation of MAPK (p38 and pJNK) in a time-dependent manner, particularly after 30 min of exposure. To further understand the contribution of MAPK pathways to IL-6 induction by IL-1β, HT-29 cells were pre-treated with specific inhibitors. As Figure 3B indicates, both the p38 inhibitor (SB) and the JNK inhibitor (SP) effectively blocked IL-1β-induced IL-6 expression in a dose-dependent manner, whereas the ERK inhibitor (PD) did not impact IL-6 expression. These observations suggest the involvement of p38 MAPK and JNK pathways in the cascade leading to IL-1β-induced IL-6 expression, while the Erk pathway seems not to be significantly involved. The pivotal roles of p38 and JNK signaling were further validated using a dominant-negative mutant MAPK expression plasmid (DN-p38, TAM, K97). Co-transfection with pGL3-IL-6 in HT-29 cells markedly reduced IL-1β-induced IL-6 promoter activity, underscoring the significance of p38 and JNK pathways in this context (Figure 3C). Subsequently, the study probed how SFN impedes IL-6 production by examining MAPK phosphorylation changes. Figure 3D shows that IL-1β treatment markedly increased p38 and JNK phosphorylation, which SFN then inhibited in a dose-responsive manner. Notably, SFN selectively inhibited the activation of p-P38 without affecting P-ERK and P-JNK.

### 3.4. SFN Blocks IL-1β-Induced Activation of the AP-1 Transcription Factor

**Figure 4:**
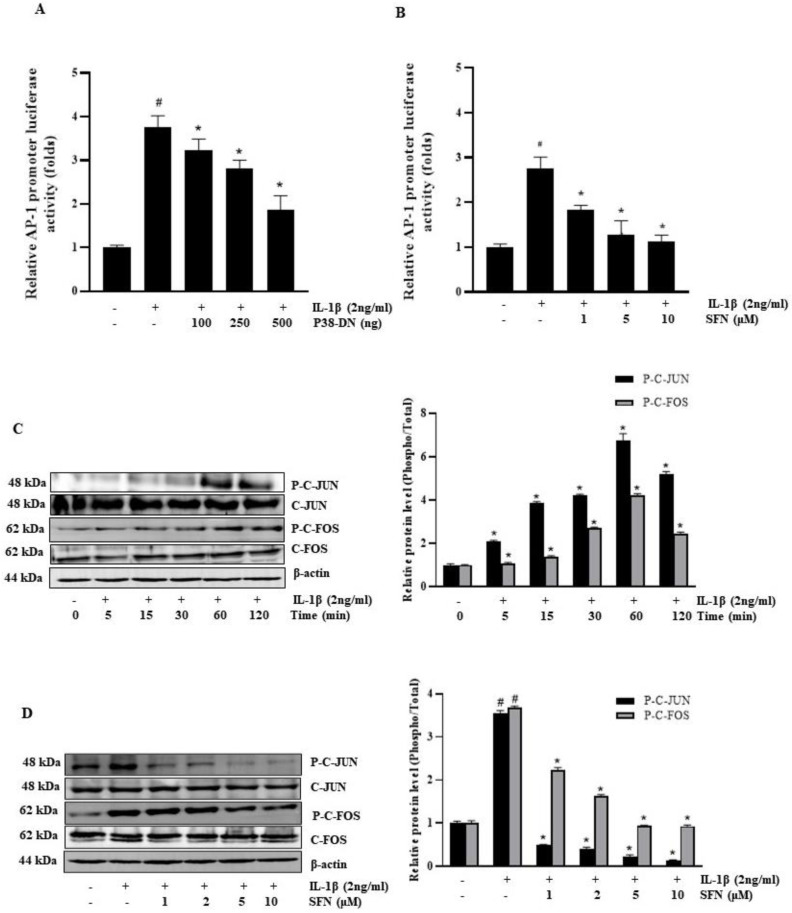
Sulforaphane inhibits IL-1β-induced IL-6 expression by suppressing the transcriptional activity of AP-1. (**A**) HT-29 cells were cotransfected with 100–500 ng of p38 MAPK dominant-negative mutant plasmid (p38-DN) and 250 ng of pGL3-AP-1 promoter construct. After a 12-h incubation with 2 ng/mL IL-1β, luciferase assays were performed on these cells. (**B**) Cells pre-treated with sulforaphane (1, 5, and 10 μM) and transiently transfected with the AP-1 reporter plasmid were incubated with 2 ng/mL IL-1β for 12 h, followed by determination of luciferase activity. (**C**) Following 0 to 120 min incubation with 2 ng/mL IL-1β, the levels of total and phosphorylated c-Fos and c-Jun were assessed by Western blotting. (**D**) Cells pre-treated with 0–10 μM sulforaphane for 1 h and then incubated with 2 ng/mL IL-1β for 1 h were analyzed for phosphorylated C-Fos and c-Jun levels via Western blot. Significance markers: # *p* < 0.05 versus control; * *p* < 0.05 versus IL-1β. Data represent mean ± SD from triplicate experiments.

Assessing the presence of IL-1β-responsive cis-regulatory elements in the IL-6 promoter is vital as they influence gene expression by altering transcription factors through signaling molecules. This study revealed that IL-1β enhances AP-1 promoter activity. However, co-transfection with the AP-1 promoter plasmid and the p38-DN plasmid (a dominant-negative mutant of p38 MAPK) significantly inhibited this IL-1β-induced activation of the AP-1 promoter (Figure 4A). Exploring their impact on SFN-mediated suppression of IL-1β expression, we observed that SFN dose-dependently reduced IL-1β-activated AP-1 in AP-1-dependent transcription studies (Figure 4B). Additionally, our results showed that IL-1β raised the levels of phosphorylated c-Fos and c-Jun (components of the AP-1 transcription factor) at different treatment intervals (Figure 4C), both of which were markedly reduced by SFN (Figure 4D).

### 3.5. SFN Inhibits the Production of ROS Triggered by IL-1β

**Figure 5:**
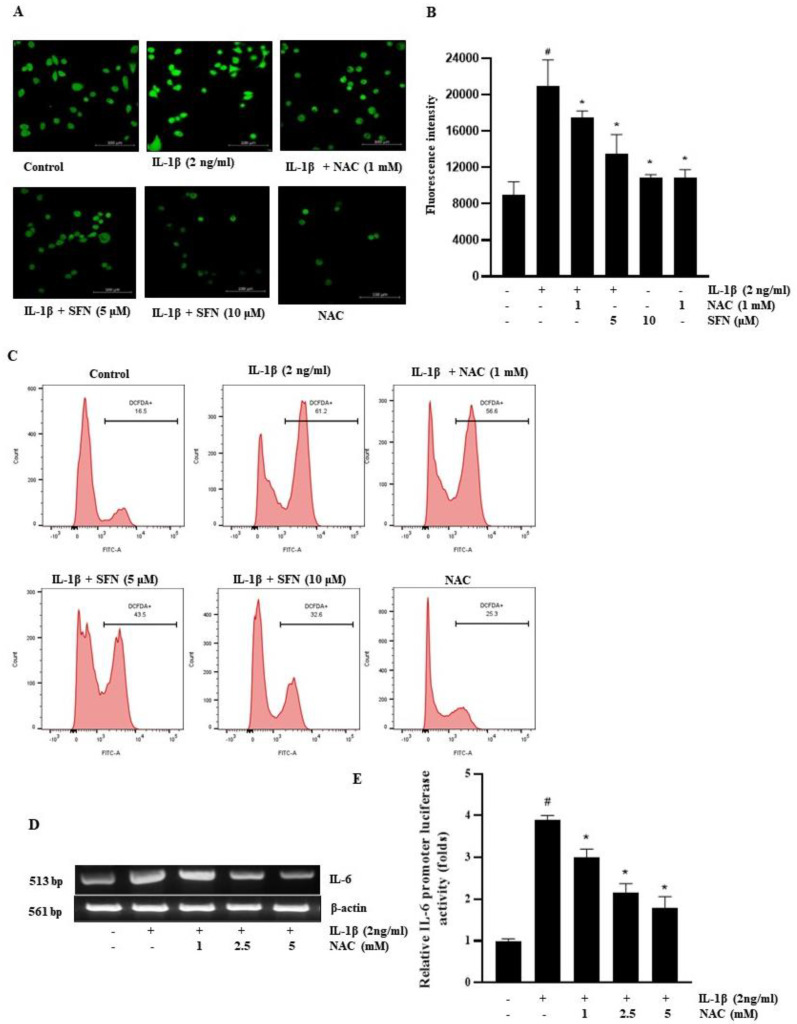
Sulforaphane inhibits the expression of IL-6 in HT-29 cells by inhibiting the production of ROS. (**A**,**B**) Confocal microscopy images (200× magnification) showing ROS production and quantitative analysis of ROS levels; scale bar: 100 µM. (**C**) Flow cytometry was employed to analyze ROS production in HT-29 cells treated with IL-1β (2 ng/mL) and pre-treated with SFN and NAC. (**D**) Cells pre-treated with 0–5 mM NAC for 1 h and then incubated with 2 ng/mL IL-1β for 4 h were subjected to mRNA extraction and RT-PCR for IL-6 mRNA expression level assessment. (**E**) Following transient transfection with an IL-6-promoter reporter plasmid, cells were pre-treated with various concentrations of NAC for 1 h and exposed to 2 ng/mL IL-1β for 12 h. Passive lysis buffer was then applied to the lysed cells to determine luciferase activity. Significance markers: # *p* < 0.05 versus control; * *p* < 0.05 versus IL-1β. Data represent mean ± SD from triplicate experiments.

To examine the role of SFN in attenuating ROS-mediated IL-1β-stimulated IL-6 expression, we employed the DCFDA assay for measuring ROS production in HT-29 cells pre-treated with SFN and IL-1β. Results indicated that IL-1β significantly induced ROS production, which was completely counteracted by pre-treatment with SFN and NAC (a ROS scavenger) (Figure 5A,B). Fluorescence-activated cell sorting (FACS) analysis further revealed that pre-treatment with SFN and NAC substantially reduced the ROS levels induced by IL-1β in HT-29 cells (Figure 5C). Additionally, NAC pre-treatment consistently and dose-dependently suppressed IL-1β-induced IL-6 expression, as shown by both RT-PCR (Figure 5C) and IL-6 promoter assays (Figure 5D). These results clearly demonstrate that ROS plays a role in IL-1β-induced IL-6 expression in HT-29 cells, and this effect can be mitigated by both NAC and SFN.

### 3.6. SFN Inhibits IL-1Β-Induced IL-6 Expression via STAT3 Signaling

**Figure 6:**
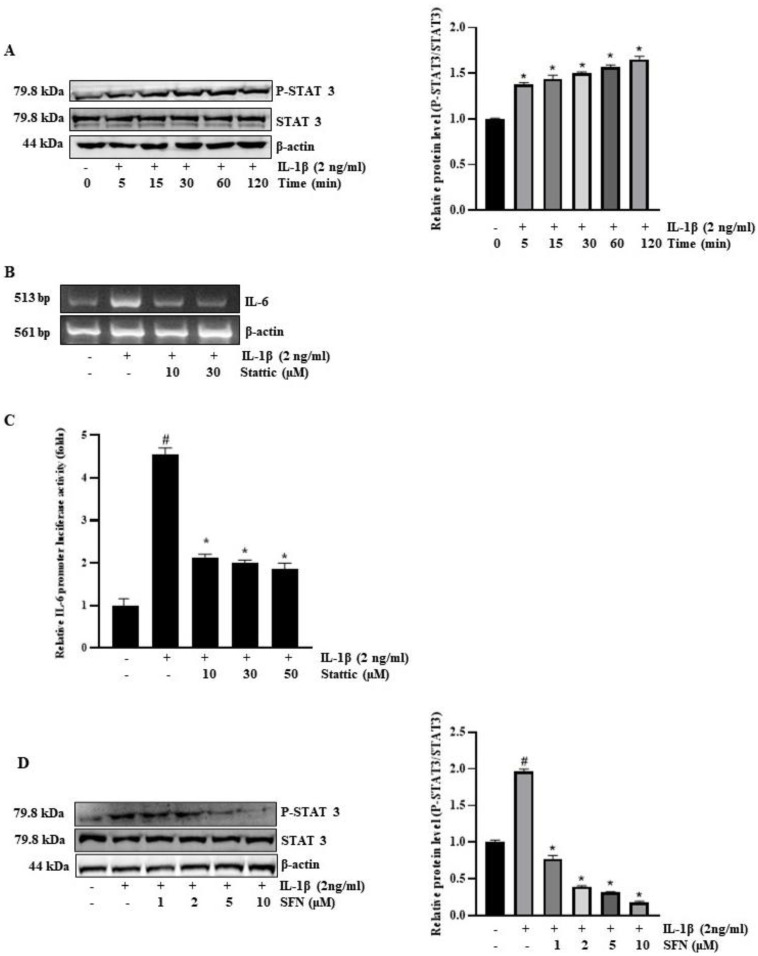
Role of STAT-3 in sulforaphane inhibition of IL-1β-induced IL-6 expression in HT-29 cells. (**A**) Cells were incubated with 2 ng/mL IL-1β for 0–120 min, followed by Western blot analysis to determine levels of total and phosphorylated STAT-3. (**B**) Pre-treatment of HT-29 cells with Stattic (10, 30 µM) for 1 h was followed by treatment with IL-1β (2 ng/mL) for 4 h, and IL-6 mRNA levels were then assessed by reverse transcription PCR. (**C**) Cells transiently transfected with 1 µg of pGL3-IL-6-promoter reporter construct and pre-treated with Stattic for 1 h were exposed to 2 ng/mL IL-1β for 12 h before luciferase activity was measured using a luminometer. (**D**) Cells pre-treated with sulforaphane for 1 h and then treated with 2 ng/mL IL-1β for 30 min were analyzed for phosphorylated STAT3 levels by Western blot. Significance markers: # *p* < 0.05 versus control; * *p* < 0.05 versus IL-1Β. Data represent mean ± SD from triplicate experiments.

To elucidate the role of STAT3 in regulating IL-6 expression, we investigated the STAT3 pathway in interleukin-mediated signaling. Following a 30-min exposure to IL-1β, we observed a significant increase in phosphorylated STAT3 (Figure 6A). Moreover, Stattic, a targeted inhibitor of STAT3 signaling, effectively reversed the IL-1β-induced augmentation of IL-6 mRNA levels and promoter activity (Figure 6B,C). We also examined whether SFN could impede the IL-1β-stimulated phosphorylation of STAT3. For this, HT-29 cells were pre-treated with SFN for one hour and subsequently exposed to IL-1β for 30 min, as depicted in Figure 6D. Our findings revealed that SFN effectively inhibited the IL-1β-stimulated phosphorylation of STAT3, corroborating our hypothesis.

### 3.7. SFN Inhibits ROS Production Stimulated STAT-3 Activation in IL-1β-Induced IL-6 Expression Mechanism

**Figure 7:**
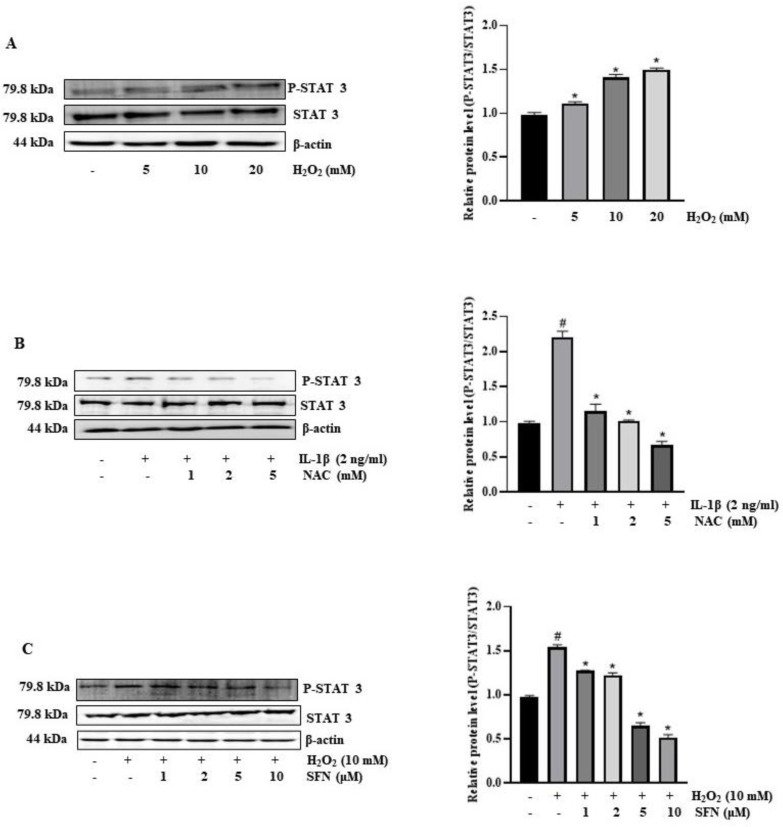
Sulforaphane’s effect on ROS production and STAT-3 activation in IL-1β-induced IL-6 expression in HT-29 cells. (**A**) Cells exposed to hydrogen peroxide (H_2_O_2_) at 0 to 20 mM for 30 min were processed for Western blot analysis to determine phosphorylated STAT3 levels. Significance markers: * *p* < 0.05 versus control. (**B**) Cells pre-treated with 0 to 10 µM sulforaphane before a 30-min exposure to H_2_O_2_ were analyzed post-incubation for phosphorylated STAT3 levels by Western blot. Significance markers: # *p* < 0.05 versus control; * *p* < 0.05 versus IL-1Β. Data represent mean ± SD from triplicate experiments. (**C**) Pre-treatment with NAC at 1 to 5 mM for 1 h before a 15-min exposure to 20 mM H_2_O_2_ was followed by protein extraction and Western blot analysis to assess phosphorylation levels of STAT3. Significance markers: # *p* < 0.05 versus control; * *p* < 0.05 versus H_2_O_2_. Data represent mean ± SD from triplicate experiments.

Our hypothesis posited that SFN mitigates IL-1β-induced IL-6 expression by inhibiting STAT3 activation, which is stimulated by ROS production. Intriguingly, exposing HT-29 cells to H_2_O_2_ resulted in a dose-dependent activation of STAT3 signaling, as evidenced in Figure 7A. We tested this hypothesis by assessing STAT3 phosphorylation levels during IL-1β treatment with and without SFN and NAC pre-treatment. Our data indicated that both SFN and NAC successfully prevented the IL-1β-induced STAT3 activation in a dose-dependent manner (Figure 7B,C). These results suggest that ROS production in response to IL-1β activates STAT3 signaling, leading to increased IL-6 expression, which is inhibited by SFN.

### 3.8. Effect of SFN on Invasiveness of HT-29 Cells

**Figure 8:**
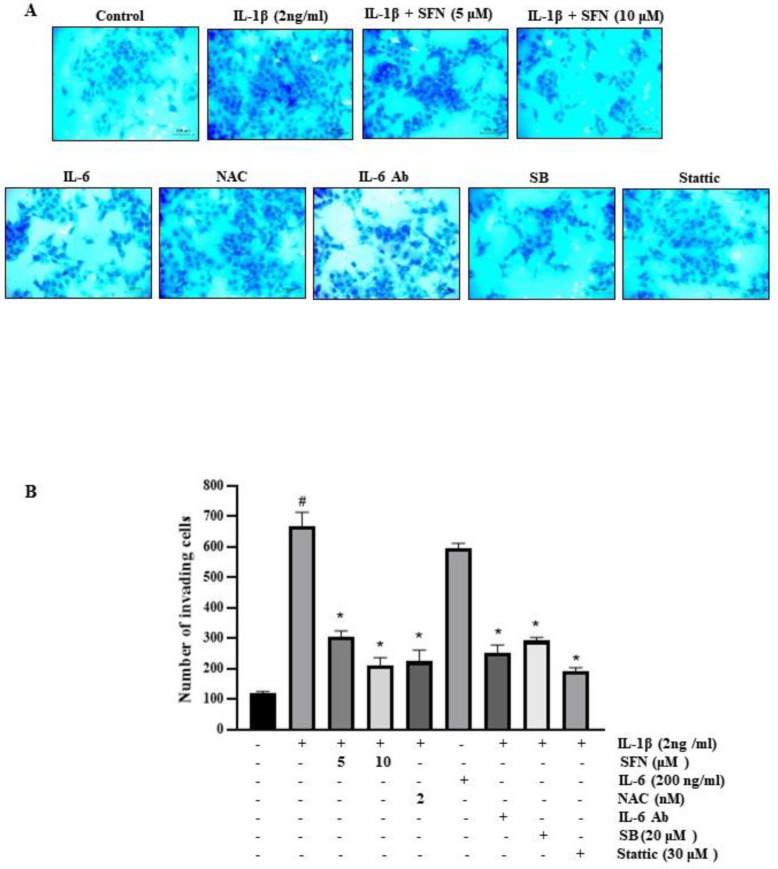
Sulforaphane inhibits the invasion of HT-29 cells by suppressing IL-1β-induced IL-6 expression. (**A**) HT-29 cells were treated with sulforaphane or anti-IL-6 antibody at 200 ng/mL, along with 2 ng/mL IL-1β in a Matrigel apparatus for 24 h. Cell invasion was monitored using a phase-contrast light microscope at 20× magnification (Scale—100 μm). (**B**) Invaded cells were counted indirectly using Diff-Quick stain, which aided in visualizing cells on the undersurface of the chamber membrane. Significance markers: # *p* < 0.05 versus control; * *p* < 0.05 versus IL-1β. Data represent mean ± SD from triplicate experiments.

In a modified Boyden invasion chamber assay, IL-6 was found to correlate with an increased invasion of cancer cells. Figure 8A demonstrates that, relative to untreated cells, a 24-h treatment with either IL-1β or IL-6 enhanced the invasive capacity of HT-29 cells through Matrigel. Subsequently, we assessed the impact of SFN and other factors on HT-29 cell invasiveness 24 h following IL-1β treatment. This involved pre-treating the cells with SFN, anti-IL-6 antibodies, SB, NAC, and Stattic. Our results indicate that SFN effectively inhibited IL-1β-induced cell invasion. Furthermore, these findings suggest that SFN attenuates the IL-1β-induced upregulation of IL-6 expression, likely by interfering with the p-38/AP-1 and ROS/STAT3 signaling pathways.

## 4. Discussion

Colon cancer remains a leading cause of death globally for both men and women, with tumor recurrence observed in nearly half of the patients with CRC [36]. Despite significant advancements over recent decades, conventional oncology treatments still encounter major challenges, including drug resistance, tumor relapse, and metastasis [37]. The exploration of natural compounds and their analogs as chemopreventive agents is gaining momentum. SFN, known for its health benefits, is garnering attention in the medical field, especially for its potential protective effects against various types of cancer [38,39,40,41,42,43], cardiovascular diseases [44,45,46], neurological diseases [47,48,49,50], insulin resistance [51,52,53], obesity [54,55,56], and musculoskeletal diseases [57,58,59]. Additionally, the consumption of broccoli, brussels sprouts, cabbage, and SFN, an isothiocyanate derivative, is linked to various health benefits, including anticancer and antioxidant properties [60].

IL-1β plays a significant role in both physiological and pathological contexts. Notably, its aberrant production and signaling are intimately associated with tumor formation, growth, and metastasis in various cancers [61]. Our studies revealed that IL-1β and IL-6 levels were elevated in the CRC cell line and that SFN effectively inhibited the IL-1β-induced upregulation of IL-6. This interplay between IL-1β and IL-6 in colon cancer progression was corroborated by findings from GEPIA and TIMER, with similar observations in a study involving African American colon cancer patients [62]. Additionally, Paredes et al. highlighted that proinflammatory cytokines, including IL-1β, IL-6, and IL-8, contribute to colon cancer development. Furthermore, Chen et al. reported increased IL-1β in C57BL/6 mice afflicted with colon cancer [63]. Recent research has also shown that SFN (20 mg/kg/day) mitigates necrotizing enterocolitis (NEC) by reducing the expression of IL-1β, IL-8, IL-10, IL-6, and TNF-α, thereby decreasing apoptosis in NEC-induced mice [64]. Gasparello J et al. found that SFN (2, 5, and 10 μM) curtails IL-6 and IL-8 expression in IB3-1 bronchial cells [65]. In a separate study, SFN-treated (1.5 mM SFN (200 μL of SFN at 12.8 mg/mL/kg)) CIA mice exhibited reduced levels of IL-6, IL-17, TNF-α, receptor activator of NF-κB ligand, and tartrate-resistant acid phosphatase [66]. Moreover, in human WM115 and WM266-4 melanoma cells, the combination of SFN (5 and 10 μM) and Fernblock^®^ XP (FB) hindered melanoma cell migration in vitro and curbed the production of MMP-1, -2, -3, and -9, inflammasome activation, and IL-1β secretion [67].

In the context of cancer chemoprevention and chemotherapy, the AP-1 and MAPK signaling pathways play a crucial role in tumor cell growth, proliferation, apoptosis, and survival [68]. Our research demonstrated that the signaling of p38 MAPK, AP-1, and STAT3 is upregulated in HT-29 cells induced by IL-1β. This finding was further substantiated using specific inhibitors (SB, SP, and PD), while SFN treatment hindered the signaling of p38 MAPK/AP-1 and STAT3. The tumor microenvironment significantly influences the expression of membrane-bound complement regulatory proteins (mCRPs) in tumor cells, contributing to tumor immune evasion [69,70]. Several studies have established that mCRPs are highly expressed in colon cancer [71,72,73]. The overexpression of mCRPs in colon cancer leads to the upregulation of STAT3/STAT6/p38 MAPK signaling [74]. Additionally, IL-1β activates miR-146a, which in turn targets p38, ERK, and JNK MAP kinases, along with downstream transcription factors GATA2, c-Fos, and c-Jun, that are implicated in metastatic progression in colon cancer cells [75]. Clinical trials have shown that SFN (200 µmol) modulates STAT3 in cancer cells, thereby preventing skin cancer and melanoma caused by ultraviolet light [76]. In previous research, we showed that IL-1β induces the upregulation of AP-1 signaling in bladder cancer cell lines [77,78]. Furthermore, Lei Gao et al. (2021) found that SFN (25 µmol/L) inhibited MAPK signaling in the SW480 colon cancer cell line [79]. Nivedita Banerjee et al. reported that treatment with SFN (8 mg/kg) reduced the trichloroethene (TCE)-induced phosphorylation of MAPK (p38, ERK, and JNK) in MRL+/+ mice [80]. Additionally, in head and neck squamous cell carcinoma, SFN (6 μmol/L) was found to promote NRF2-independent dephosphorylation/inactivation of pSTAT3 in 4-nitroquinoline-1-oxide (4NQO)-induced C57BL/6 mice [81]. Other in vivo studies have demonstrated that SFN (1–50 μM) obstructs ROS-mediated p38/AP-1 signaling in nicotine-induced gastric cancer cell lines [43].

**Figure 9:**
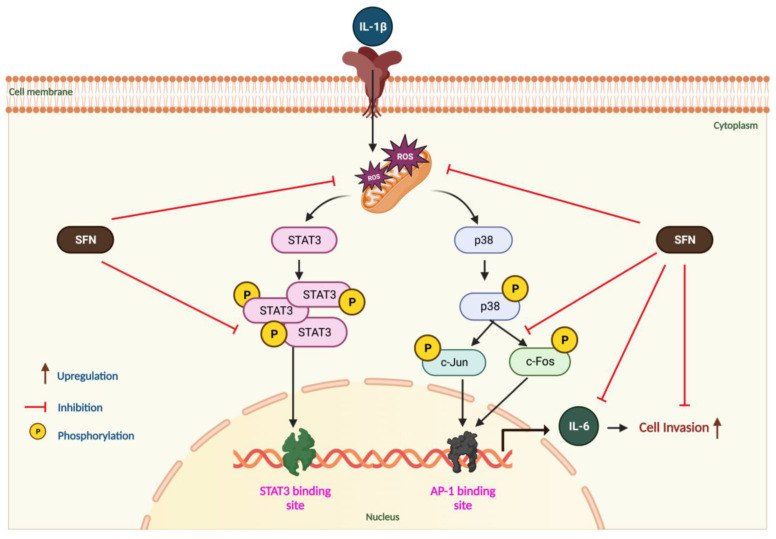
Schematic diagram of SFN-mediated inhibition of IL-1β-induced IL-6 expression in CRC cells. The diagram illustrates the process where IL-1β-induced ROS activates MAPK (p38 and JNK) and STAT3 pathways. This activation leads p38 to stimulate c-Jun, binding to AP-1 and STAT3 binding sites, thereby triggering IL-6 expression and increasing the invasiveness of CRC cells. SFN counters this effect by inhibiting ROS production, thus suppressing IL-1β-induced IL-6 expression.

Multiple antioxidants maintain the balance of ROS in healthy tissues, ensuring redox homeostasis. However, strong evidence suggests that excessive oxidative reactions, resulting from a dysfunctional redox system, lead to damage in DNA, proteins, and lipids. This damage is implicated in the initiation, progression, and metastasis of cancer [82]. Studies have established that proinflammatory cytokines, such as IL-1β, trigger the generation of ROS both intracellularly and extracellularly in various in vivo and in vitro models [83,84,85]. A significant study on hypoxic pulmonary hypertension by Jinjin Pan et al. (2023) demonstrated that SFN (2 mg/kg) enhanced serum superoxide dismutase (SOD) activity, SOD2 expression, and total glutathione levels, while also increasing the GSH/GSSG ratio in pulmonary artery smooth muscle cells (PASMCs). This study also noted a reduction in malondialdehyde (MDA) levels in serum and ROS production in PASMCs [44]. Additionally, our data revealed that IL-1β-treated cells showed increased invasiveness, an effect mitigated by SFN treatment. IL-1β may foster CRC growth and invasion by stimulating colon cancer stem cell (CSC) self-renewal and upregulating stemness factor genes Bmi1 and Nestin. EMT, Zub1, and enhanced drug resistance are known to play significant roles in these processes [86]. SFN (5 and 10 µM) was found to reduce viability and induce apoptosis in HCT116 and RKO CRC cells. It also brought about epigenetic changes in these cells by downregulating HDAC1 and hTERT mRNA expression, with hTERT being crucial for constant proliferation, EMT, and stemness traits in cancer cells [87,88]. In summary, IL-1β-induced cancer growth, ROS production, and cell invasiveness are mediated through the regulation of MAPK pathways (p38, ERK, JNK) and their downstream transcription factors c-Fos, c-Jun, and STAT3 in HT-29 cells, effects all inhibited by SFN, as illustrated in our schematic diagram (Figure 9). Our findings confirm that IL-1β induced IL-6, which in turn upregulated ROS-mediated MAPK/AP-1/STAT3 signaling. Furthermore, SFN displayed significant antioxidant and chemopreventive activities by inhibiting the IL-1β-induced expression of IL-6 and the subsequent MAPK/AP-1/STAT3 signaling in HT-29 colon cancer cells.

## 5. Conclusions

Considering the significant disease burden associated with CRC, it is essential to identify effective biomarkers for predicting prognosis and determining the optimal therapeutic strategy. Sulforaphane, a naturally occurring compound, plays a critical role in preventing CRC development. It achieves this by inhibiting oxidative stress, downregulating the MAPK/AP-1/STAT3 signaling pathway, and suppressing cell invasion. Therefore, the anticancer properties of SFN, along with its potential as a chemopreventive agent, warrant additional clinical research.

## Data Availability

All data are contained within the article.

## Supplementary Materials

The following supporting information can be downloaded at: https://www.mdpi.com/article/10.3390/antiox13040406/s1, Table S1: The list of primers used in this study.

## References

[1] Hossain M.S., Karuniawati H., Jairoun A.A., Urbi Z., Ooi D.J., John A., Lim Y.C., Kibria K.K., Mohiuddin A., Ming L.C. (2022). Colorectal cancer: A review of carcinogenesis, global epidemiology, current challenges, risk factors, preventive and treatment strategies. Cancers.

[2] Douaiher J., Ravipati A., Grams B., Chowdhury S., Alatise O., Are C. (2017). Colorectal cancer—Global burden, trends, and geographical variations. J. Surg. Oncol..

[3] Alzahrani S.M., Al Doghaither H.A., Al-Ghafari A.B. (2021). General insight into cancer: An overview of colorectal cancer. Mol. Clin. Oncol..

[4] Heavey P.M., McKenna D., Rowland I.R. (2004). Colorectal cancer and the relationship between genes and the environment. Nutr. Cancer.

[5] Lucafò M., Curci D., Franzin M., Decorti G., Stocco G. (2021). Inflammatory bowel disease and risk of colorectal cancer: An overview from pathophysiology to pharmacological prevention. Front. Pharmacol..

[6] Karunanithi S., Levi L., DeVecchio J., Karagkounis G., Reizes O., Lathia J.D., Kalady M.F., Noy N. (2017). RBP4-STRA6 pathway drives cancer stem cell maintenance and mediates high-fat diet-induced colon carcinogenesis. Stem Cell Rep..

[7] O’Neill A.M., Burrington C.M., Gillaspie E.A., Lynch D.T., Horsman M.J., Greene M.W. (2016). High-fat Western diet–induced obesity contributes to increased tumor growth in mouse models of human colon cancer. Nutr. Res..

[8] Lin T.-C., Chien W.-C., Hu J.-M., Tzeng N.-S., Chung C.-H., Pu T.-W., Hsiao C.-W., Chen C.-Y. (2020). Risk of colorectal cancer in patients with alcoholism: A nationwide, population-based nested case-control study. PLoS ONE.

[9] Zhao H., Chen D., Cao R., Wang S., Yu D., Liu Y., Jiang Y., Xu M., Luo J., Wang S. (2018). Alcohol consumption promotes colorectal carcinoma metastasis via a CCL5-induced and AMPK-pathway-mediated activation of autophagy. Sci. Rep..

[10] Kastrinos F., Samadder N.J., Burt R.W. (2020). Use of family history and genetic testing to determine risk of colorectal cancer. Gastroenterology.

[11] Wark P.A., Wu K., Van’t Veer P., Fuchs C.F., Giovannucci E.L. (2009). Family history of colorectal cancer: A determinant of advanced adenoma stage or adenoma multiplicity?. Int. J. Cancer.

[12] Abu-Remaileh M., Bender S., Raddatz G., Ansari I., Cohen D., Gutekunst J., Musch T., Linhart H., Breiling A., Pikarsky E. (2015). Chronic Inflammation Induces a Novel Epigenetic Program That Is Conserved in Intestinal Adenomas and in Colorectal CancerDNA Methylation Links Inflammation and Cancer. Cancer Res..

[13] Kanehara K., Ohnuma S., Kanazawa Y., Sato K., Kokubo S., Suzuki H., Karasawa H., Suzuki T., Suzuki C., Naitoh T. (2019). The indole compound MA-35 attenuates tumorigenesis in an inflammation-induced colon cancer model. Sci. Rep..

[14] Singh N., Baby D., Rajguru J.P., Patil P.B., Thakkannavar S.S., Pujari V.B. (2019). Inflammation and cancer. Ann. Afr. Med..

[15] Zhao H., Wu L., Yan G., Chen Y., Zhou M., Wu Y., Li Y. (2021). Inflammation and tumor progression: Signaling pathways and targeted intervention. Signal Transduct. Target. Ther..

[16] Colotta F., Allavena P., Sica A., Garlanda C., Mantovani A. (2009). Cancer-related inflammation, the seventh hallmark of cancer: Links to genetic instability. Carcinogenesis.

[17] Rébé C., Ghiringhelli F. (2020). Interleukin-1β and cancer. Cancers.

[18] Garon E.B., Yang J.C.-H., Dubinett S.M. (2020). The role of interleukin 1β in the pathogenesis of lung cancer. JTO Clin. Res. Rep..

[19] Filaly H.E., Outlioua A., Medyouf H., Guessous F., Akarid K. (2022). Targeting IL-1β in patients with advanced Helicobacter pylori infection: A potential therapy for gastric cancer. Future Microbiol..

[20] Schneider L., Liu J., Zhang C., Azoitei A., Meessen S., Zheng X., Cremer C., Gorzelanny C., Kempe-Gonzales S., Brunner C. (2021). The Role of Interleukin-1-Receptor-Antagonist in Bladder Cancer Cell Migration and Invasion. Int. J. Mol. Sci..

[21] Chen Y., Yang Z., Deng B., Wu D., Quan Y., Min Z. (2020). Interleukin 1β/1RA axis in colorectal cancer regulates tumor invasion, proliferation and apoptosis via autophagy. Oncol. Rep..

[22] Jung J., Lee Y.-H., Fang X., Kim S.-J., Kim S.H., Kim D.-H., Song N.-Y., Na H.-K., Baek J.-H., Surh Y.-J. (2021). IL-1β induces expression of proinflammatory cytokines and migration of human colon cancer cells through upregulation of SIRT1. Arch. Biochem. Biophys..

[23] Van Cutsem E., Shitara K., Deng W., Vaury A., Tseng L., Wang X., Millholland J., Shilkrut M., Mookerjee B., Jonasch E. (2019). Gevokizumab, an interleukin-1β (IL-1β) monoclonal antibody (mAb), in metastatic colorectal cancer (mCRC), metastatic gastroesophageal cancer (mGEC) and metastatic renal cell carcinoma (mRCC):“First-in-cancer” phase Ib study. Ann. Oncol..

[24] Waldner M.J., Foersch S., Neurath M.F. (2012). Interleukin-6-a key regulator of colorectal cancer development. Int. J. Biol. Sci..

[25] Lin Y., He Z., Ye J., Liu Z., She X., Gao X., Liang R. (2020). Progress in understanding the IL-6/STAT3 pathway in colorectal cancer. OncoTargets Ther..

[26] Shi W., Men L., Pi X., Jiang T., Peng D., Huo S., Luo P., Wang M., Guo J., Jiang Y. (2021). Shikonin suppresses colon cancer cell growth and exerts synergistic effects by regulating ADAM17 and the IL-6/STAT3 signaling pathway. Int. J. Oncol..

[27] Liu J., Liu Y., Chen J., Hu C., Teng M., Jiao K., Shen Z., Zhu D., Yue J., Li Z. (2017). The ROS-mediated activation of IL-6/STAT3 signaling pathway is involved in the 27-hydroxycholesterol-induced cellular senescence in nerve cells. Toxicol. Vitr..

[28] Costa-Pereira A.P. (2014). Regulation of IL-6-type cytokine responses by MAPKs. Biochem. Soc. Trans..

[29] Cahill C.M., Rogers J.T. (2008). Interleukin (IL) 1β induction of IL-6 is mediated by a novel phosphatidylinositol 3-kinase-dependent AKT/IκB kinase α pathway targeting activator protein-1. J. Biol. Chem..

[30] Ishida K., Kaji K., Sato S., Ogawa H., Takagi H., Takaya H., Kawaratani H., Moriya K., Namisaki T., Akahane T. (2021). Sulforaphane ameliorates ethanol plus carbon tetrachloride-induced liver fibrosis in mice through the Nrf2-mediated antioxidant response and acetaldehyde metabolization with inhibition of the LPS/TLR4 signaling pathway. J. Nutr. Biochem..

[31] Wu G., Yan Y., Zhou Y., Duan Y., Zeng S., Wang X., Lin W., Ou C., Zhou J., Xu Z. (2020). Sulforaphane: Expected to become a novel anti-tumor compound. Oncol. Res. Featur. Preclin. Clin. Cancer Ther..

[32] Davis R., Singh K.P., Kurzrock R., Shankar S. (2009). Sulforaphane inhibits angiogenesis through activation of FOXO transcription factors. Oncol. Rep..

[33] Zhang Y.-j., Wu Q. (2021). Sulforaphane protects intestinal epithelial cells against lipopolysaccharide-induced injury by activating the AMPK/SIRT1/PGC-1α pathway. Bioengineered.

[34] Xia Y., Khoi P.N., Yoon H.J., Lian S., Joo Y.E., Chay K.O., Kim K.K., Jung Y.D. (2015). Piperine inhibits IL-1β-induced IL-6 expression by suppressing p38 MAPK and STAT3 activation in gastric cancer cells. Mol. Cell. Biochem..

[35] Li S., Nguyen T.T., Ung T.T., Sah D.K., Park S.Y., Lakshmanan V.-K., Jung Y.D. (2022). Piperine attenuates lithocholic acid-stimulated interleukin-8 by suppressing Src/EGFR and reactive oxygen species in human colorectal cancer cells. Antioxidants.

[36] Gupta R., Bhatt L.K., Johnston T.P., Prabhavalkar K.S. (2019). Colon cancer stem cells: Potential target for the treatment of colorectal cancer. Cancer Biol. Ther..

[37] Wang D.-Y., Jiang Z., Ben-David Y., Woodgett J.R., Zacksenhaus E. (2019). Molecular stratification within triple-negative breast cancer subtypes. Sci. Rep..

[38] Han S., Wang Z., Liu J., Wang H.-M.D., Yuan Q. (2021). miR-29a-3p-dependent COL3A1 and COL5A1 expression reduction assists sulforaphane to inhibit gastric cancer progression. Biochem. Pharmacol..

[39] Iida Y., Okamoto-Κatsuyama M., Maruoka S., Mizumura K., Shimizu T., Shikano S., Hikichi M., Takahashi M., Tsuya K., Okamoto S. (2021). Effective ferroptotic small-cell lung cancer cell death from SLC7A11 inhibition by sulforaphane. Oncol. Lett..

[40] Zhang Y., Lu Q., Li N., Xu M., Miyamoto T., Liu J. (2022). Sulforaphane suppresses metastasis of triple-negative breast cancer cells by targeting the RAF/MEK/ERK pathway. NPJ Breast Cancer.

[41] Huang L., He C., Zheng S., Wu C., Ren M., Shan Y. (2022). AKT1/HK2 Axis-mediated Glucose Metabolism: A Novel Therapeutic Target of Sulforaphane in Bladder Cancer. Mol. Nutr. Food Res..

[42] Huang B., Lei S., Wang D., Sun Y., Yin J. (2021). Sulforaphane exerts anticancer effects on human liver cancer cells via induction of apoptosis and inhibition of migration and invasion by targeting MAPK7 signalling pathway. J. BUON.

[43] Li S., Khoi P.N., Yin H., Sah D.K., Kim N.-H., Lian S., Jung Y.-D. (2022). Sulforaphane Suppresses the Nicotine-Induced Expression of the Matrix Metalloproteinase-9 via Inhibiting ROS-Mediated AP-1 and NF-κB Signaling in Human Gastric Cancer Cells. Int. J. Mol. Sci..

[44] Pan J., Wang R., Pei Y., Wang D., Wu N., Ji Y., Tang Q., Liu L., Cheng K., Liu Q. (2023). Sulforaphane alleviated vascular remodeling in hypoxic pulmonary hypertension via inhibiting inflammation and oxidative stress. J. Nutr. Biochem..

[45] Sun Y., Zhou S., Guo H., Zhang J., Ma T., Zheng Y., Zhang Z., Cai L. (2020). Protective effects of sulforaphane on type 2 diabetes-induced cardiomyopathy via AMPK-mediated activation of lipid metabolic pathways and NRF2 function. Metabolism.

[46] Poletto Bonetto J.H., Luz de Castro A., Fernandes R.O., Corssac G.B., Cordero E.A., Schenkel P.C., Sander da Rosa Araujo A., Belló-Klein A. (2022). Sulforaphane Effects on Cardiac Function and Calcium-Handling–Related Proteins in 2 Experimental Models of Heart Disease: Ischemia-Reperfusion and Infarction. J. Cardiovasc. Pharmacol..

[47] Kim J. (2021). Pre-clinical neuroprotective evidences and plausible mechanisms of Sulforaphane in Alzheimer’s disease. Int. J. Mol. Sci..

[48] Yang C., Qin S., Zhang J., Wang Y., Li H., Lü T. (2022). Sulforaphane Upregulates Cultured Mouse Astrocytic Aquaporin-4 Expression through p38 MAPK Pathway. J. Healthc. Eng..

[49] Cao Q., Zou Q., Zhao X., Zhang Y., Qu Y., Wang N., Murayama S., Qi Q., Hashimoto K., Lin S. (2022). Regulation of BDNF transcription by Nrf2 and MeCP2 ameliorates MPTP-induced neurotoxicity. Cell Death Discov..

[50] Bai X., Bian Z., Zhang M. (2022). Targeting the Nrf2 signaling pathway using phytochemical ingredients: A novel therapeutic road map to combat neurodegenerative diseases. Phytomedicine.

[51] Teng W., Li Y., Du M., Lei X., Xie S., Ren F. (2019). Sulforaphane prevents hepatic insulin resistance by blocking serine palmitoyltransferase 3-mediated ceramide biosynthesis. Nutrients.

[52] Zhang Y., Wu Q., Liu J., Zhang Z., Ma X., Zhang Y., Zhu J., Thring R.W., Wu M., Gao Y. (2022). Sulforaphane alleviates high fat diet-induced insulin resistance via AMPK/Nrf2/GPx4 axis. Biomed. Pharmacother..

[53] Tian S., Wang Y., Li X., Liu J., Wang J., Lu Y. (2021). Sulforaphane regulates glucose and lipid metabolisms in obese mice by restraining JNK and activating insulin and FGF21 signal pathways. J. Agric. Food Chem..

[54] Sun Y., Tang Z., Hao T., Qiu Z., Zhang B. (2022). Simulated Digestion and Fermentation In Vitro by Obese Human Gut Microbiota of Sulforaphane from Broccoli Seeds. Foods.

[55] Işın Ç., Pauline L.P., Hadley C.K., El-Gamal A., Amina F., Dina E., Mohamed O., Rizk N.M., Masoud G.-L. (2022). Sulforaphane reduces obesity by reversing leptin resistance. eLife.

[56] Ranaweera S.S., Natraj P., Rajan P., Dayarathne L.A., Mihindukulasooriya S.P., Dinh D.T.T., Jee Y., Han C.-H. (2022). Anti-obesity effect of sulforaphane in broccoli leaf extract on 3T3-L1 adipocytes and ob/ob mice. J. Nutr. Biochem..

[57] Gambari L., Barone M., Amore E., Grigolo B., Filardo G., Iori R., Citi V., Calderone V., Grassi F. (2022). Glucoraphanin increases intracellular hydrogen sulfide (H2S) levels and stimulates osteogenic differentiation in human mesenchymal stromal cell. Nutrients.

[58] Chen M., Huang L., Lv Y., Li L., Dong Q. (2021). Sulforaphane protects against oxidative stress-induced apoptosis via activating SIRT1 in mouse osteoarthritis. Mol. Med. Rep..

[59] Du Y., Wang Q., Tian N., Lu M., Zhang X.-L., Dai S.-M. (2020). Knockdown of Nrf2 exacerbates TNF-α-induced proliferation and invasion of rheumatoid arthritis fibroblast-like synoviocytes through activating JNK pathway. J. Immunol. Res..

[60] Vanduchova A., Anzenbacher P., Anzenbacherova E. (2019). Isothiocyanate from broccoli, sulforaphane, and its properties. J. Med. Food.

[61] Pretre V., Papadopoulos D., Regard J., Pelletier M., Woo J. (2022). Interleukin-1 (IL-1) and the inflammasome in cancer. Cytokine.

[62] Paredes J., Zabaleta J., Garai J., Ji P., Imtiaz S., Spagnardi M., Alvarado J., Li L., Akadri M., Barrera K. (2020). Immune-related gene expression and cytokine secretion is reduced among African American colon cancer patients. Front. Oncol..

[63] Chen H., Ye C., Cai B., Zhang F., Wang X., Zhang J., Zhang Z., Guo Y., Yao Q. (2022). Berberine inhibits intestinal carcinogenesis by suppressing intestinal pro-inflammatory genes and oncogenic factors through modulating gut microbiota. BMC Cancer.

[64] Wang X., Mi Y., Xiong X., Bao Z. (2023). The protective effect of sulforaphane on ER-induced apoptosis and inflamamiton in necrotizing enterocolitis mice. Comb. Chem. High Throughput Screen..

[65] Gasparello J., D’Aversa E., Papi C., Gambari L., Grigolo B., Borgatti M., Finotti A., Gambari R. (2021). Sulforaphane inhibits the expression of interleukin-6 and interleukin-8 induced in bronchial epithelial IB3-1 cells by exposure to the SARS-CoV-2 Spike protein. Phytomedicine.

[66] Moon S.-J., Jhun J., Ryu J., Kwon J.y., Kim S.-Y., Jung K., Cho M.-L., Min J.-K. (2021). Correction: The anti-arthritis effect of sulforaphane, an activator of Nrf2, is associated with inhibition of both B cell differentiation and the production of inflammatory cytokines. PLoS ONE.

[67] Serini S., Guarino R., Ottes Vasconcelos R., Celleno L., Calviello G. (2020). The Combination of Sulforaphane and Fernblock^®^ XP Improves Individual Beneficial Effects in Normal and Neoplastic Human Skin Cell Lines. Nutrients.

[68] Xu C., Shen G., Yuan X., Kim J.-h., Gopalkrishnan A., Keum Y.-S., Nair S., Kong A.-N.T. (2006). ERK and JNK signaling pathways are involved in the regulation of activator protein 1 and cell death elicited by three isothiocyanates in human prostate cancer PC-3 cells. Carcinogenesis.

[69] Montalvo-Castro R.E., Salinas-Jazmín N. (2022). Relationship between the expression of complement inhibitory proteins and therapeutic efficacy of antibodies in breast cancer. Gac. Médica De México.

[70] Geller A., Yan J. (2019). The role of membrane bound complement regulatory proteins in tumor development and cancer immunotherapy. Front. Immunol..

[71] Shang Y., Chai N., Gu Y., Ding L., Yang Y., Zhou J., Ren G., Hao X., Fan D., Wu K. (2014). Systematic immunohistochemical analysis of the expression of CD46, CD55, and CD59 in colon cancer. Arch. Pathol. Lab. Med..

[72] Wu Y., Wang Y., Qin F., Wang Z., Wang Y., Yang Y., Zheng H., Wang Y. (2014). CD55 limits sensitivity to complement-dependent cytolysis triggered by heterologous expression of α-gal xenoantigen in colon tumor cells. Am. J. Physiol.-Gastrointest. Liver Physiol..

[73] Fan Y., Liao J., Wang Y., Wang Z., Zheng H., Wang Y. (2023). miR-132-3p regulates antibody-mediated complement-dependent cytotoxicity in colon cancer cells by directly targeting CD55. Clin. Exp. Immunol..

[74] Tang G., Pan L., Wang Z., Zhu H., Yang Y., Wang Z., Yue H., Shi Y., Wu D., Jiang Z. (2023). Knockdown of membrane-bound complement regulatory proteins suppresses colon cancer growth in mice through inducing tumor cell apoptosis. Int. Immunopharmacol..

[75] Zhong L., Huot J., Simard M.J. (2018). p38 activation induces production of miR-146a and miR-31 to repress E-selectin expression and inhibit transendothelial migration of colon cancer cells. Sci. Rep..

[76] Tahata S., Singh S.V., Lin Y., Hahm E.-R., Beumer J.H., Christner S.M., Rao U.N., Sander C., Tarhini A.A., Tawbi H. (2018). Evaluation of Biodistribution of Sulforaphane after Administration of Oral Broccoli Sprout Extract in Melanoma Patients with Multiple Atypical NeviEvaluation of Sulforaphane in Patients with Atypical Nevi. Cancer Prev. Res..

[77] Sah D.K., Khoi P.N., Li S., Arjunan A., Jeong J.-U., Jung Y.D. (2022). (-)-Epigallocatechin-3-Gallate Prevents IL-1β-Induced uPAR Expression and Invasiveness via the Suppression of NF-κB and AP-1 in Human Bladder Cancer Cells. Int. J. Mol. Sci..

[78] Xia Y., Yuan M., Li S., Thuan U.T., Nguyen T.T., Kang T.W., Liao W., Lian S., Jung Y.D. (2018). Apigenin suppresses the IL-1β-induced expression of the urokinase-type plasminogen activator receptor by inhibiting MAPK-mediated AP-1 and NF-κB signaling in human bladder cancer T24 cells. J. Agric. Food Chem..

[79] Gao L., Du F., Wang J., Zhao Y., Liu J., Cai D., Zhang X., Wang Y., Zhang S. (2021). Examination of the differences between sulforaphane and sulforaphene in colon cancer: A study based on next-generation sequencing. Oncol. Lett..

[80] Banerjee N., Wang H., Wang G., Boor P.J., Khan M.F. (2021). Redox-sensitive Nrf2 and MAPK signaling pathways contribute to trichloroethene-mediated autoimmune disease progression. Toxicology.

[81] Bauman J.E., Zang Y., Sen M., Li C., Wang L., Egner P.A., Fahey J.W., Normolle D.P., Grandis J.R., Kensler T.W. (2016). Prevention of carcinogen-induced oral cancer by sulforaphane. Cancer Prev. Res..

[82] Tafani M., Sansone L., Limana F., Arcangeli T., De Santis E., Polese M., Fini M., Russo M.A. (2016). The interplay of reactive oxygen species, hypoxia, inflammation, and sirtuins in cancer initiation and progression. Oxidative Med. Cell. Longev..

[83] Yan B., Han P., Pan L., Lu W., Xiong J., Zhang M., Zhang W., Li L., Wen Z. (2014). Il-1β and reactive oxygen species differentially regulate neutrophil directional migration and basal random motility in a Zebrafish injury–induced inflammation model. J. Immunol..

[84] Yang D., Elner S.G., Bian Z.-M., Till G.O., Petty H.R., Elner V.M. (2007). Pro-inflammatory cytokines increase reactive oxygen species through mitochondria and NADPH oxidase in cultured RPE cells. Exp. Eye Res..

[85] Ansari M.Y., Khan N.M., Ahmad I., Haqqi T.M. (2018). Parkin clearance of dysfunctional mitochondria regulates ROS levels and increases survival of human chondrocytes. Osteoarthr. Cartil..

[86] Li Y., Wang L., Pappan L., Galliher-Beckley A., Shi J. (2012). IL-1β promotes stemness and invasiveness of colon cancer cells through Zeb1 activation. Mol. Cancer.

[87] Martin S.L., Kala R., Tollefsbol T.O. (2018). Mechanisms for the inhibition of colon cancer cells by sulforaphane through epigenetic modulation of microRNA-21 and human telomerase reverse transcriptase (hTERT) down-regulation. Curr. Cancer Drug Targets.

[88] Liu S., Cong Y., Wang D., Sun Y., Deng L., Liu Y., Martin-Trevino R., Shang L., McDermott S., Landis M. (2014). Breast cancer stem cells transition between epithelial and mesenchymal states reflective of their normal counterparts. Stem Cell Rep..

