# Supplementary material for: Sulforaphane Inhibits IL-1β-Induced IL-6 by Suppressing ROS Production, AP-1, and STAT3 in Colorectal Cancer HT-29 Cells
Source: Antioxidants (Basel). 2024 Mar 28;13(4):406. doi: 10.3390/antiox13040406 (PMC11047376; doi:10.3390/antiox13040406)
Supplement: Supplementary file 1 [file antioxidants-13-00406-s001.zip › antioxidants-2886352-supplementary.pdf]

Supplementary Data

Sulforaphane inhibits IL-1β-induced IL-6 by suppressing ROS production, AP-1, and STAT3 in colorectal cancer HT-29 cells

Dhiraj Kumar Sah[1], **Archana Arjunan**[1], **Sun Young Park**[2], **Bora Lee**[1*], **Young Do Jung**[1*]

[1] Department of Biochemistry, Chonnam National University Medical School, Hwasun 58128, South Korea

[2] Department of Internal Medicine, Chonnam National University Medical School, Gwangju 501190, South Korea

*Corresponding authors: Bora Lee, MD, PhD; Young Do Jung, MD, PhD

Department of Biochemistry, Chonnam National University Medical School, Seoyang Ro 264, Hwasun, Jeonnam 58138, Korea

Fax: 82-81-379-2781; E-mail: blee03@jnu.ac.kr (BL), ydjung@chonnam.ac.kr (YDJ)

Supplementary Table S1. The list of primers used in this study

**Gene**	**Forward primer**	**Reverse primer**	**Accession number**
β-actin	5′-AAG CAG GAG TAT GAC GAG TC-3′	5′-GCC TTC ATA CAT CTC AAG TT-3′	NM_001101.5
IL-6	5′-ACA CAG ACA GCC ACT CAC C-3′	5′-TACATTTGCCGAAGAGCC-3′	NM_001371096.1
GAPDH	5′-TGG TAT CGT GGA AGG ACT CA-3′	5′-GGA TGA TGT TCT GGA GAG CC-3′	NM_001289745.3
